# An MEG signature corresponding to an axiomatic model of reward prediction error

**DOI:** 10.1016/j.neuroimage.2011.06.051

**Published:** 2012-01-02

**Authors:** Deborah Talmi, Lluis Fuentemilla, Vladimir Litvak, Emrah Duzel, Raymond J. Dolan

**Affiliations:** a Wellcome Trust Centre for Neuroimaging, UCL, 12 Queen Square, London WC1N 3BG, UK; b School of Psychological Sciences, University of Manchester, Oxford Road, Manchester M13 9PL, UK; c Institute of Cognitive Neuroscience, UCL, 17 Queen Square, London WC1N 3AR, UK; d Institute of Biomedicine Research of Bellvitge, University of Barcelona, 08907 l'Hospitalet de Llobregat, Spain

**Keywords:** Decision-making, Prediction error, Reward, MEG, Feedback-related negativity, Error-related negativity

## Abstract

Optimal decision-making is guided by evaluating the outcomes of previous decisions. Prediction errors are theoretical teaching signals which integrate two features of an outcome: its inherent value and prior expectation of its occurrence. To uncover the magnetic signature of prediction errors in the human brain we acquired magnetoencephalographic (MEG) data while participants performed a gambling task. Our primary objective was to use formal criteria, based upon an axiomatic model (Caplin and Dean, 2008a), to determine the presence and timing profile of MEG signals that express prediction errors. We report analyses at the sensor level, implemented in SPM8, time locked to outcome onset. We identified, for the first time, a MEG signature of prediction error, which emerged approximately 320 ms after an outcome and expressed as an interaction between outcome valence and probability. This signal followed earlier, separate signals for outcome valence and probability, which emerged approximately 200 ms after an outcome. Strikingly, the time course of the prediction error signal, as well as the early valence signal, resembled the Feedback-Related Negativity (FRN). In simultaneously acquired EEG data we obtained a robust FRN, but the win and loss signals that comprised this difference wave did not comply with the axiomatic model. Our findings motivate an explicit examination of the critical issue of timing embodied in computational models of prediction errors as seen in human electrophysiological data.

## Highlights

► We identified, for the first time, an MEG signature of a human prediction error. ► The waveform resembled the Feedback-Related Negativity (FRN) signal in EEG. ► MEG effects of probability and valence were emerged before the prediction error signals, 200 ms after the outcome. ► The EEG data revealed classic FRN which was modulated by probability.

## Introduction

The way humans and animals respond to the environment depends on prior expectations about the likelihood of events, and on whether they perceive those events to be good or bad (Dickinson and Balleine, 1994). A prediction error signal is a theoretical teaching signal (Sutton and Barto, 1998) which integrates the probability and value of events, and expresses a signed discrepancy between predictions and reality. Prediction errors are positive when outcomes are better than expected and negative when outcomes are worse than expected, and differ from baseline most strongly when outcomes are least likely (Rescorla and Wagner, 1972; Sutton and Barto, 1998). The prediction error signal can be thought of as an adaptive mechanism since it optimizes choices and actions (Cohen and Ranganath, 2005; Friston, 2010; Montague and King-Casas, 2007).

Data from animal experiments indicate that the firing of dopaminergic midbrain neurons express a prediction error (Schultz et al., 1997; Schultz and Dickinson, 2000). Phasic activations of dopaminergic neurons reflect positive prediction errors while pauses in firing reflect negative prediction errors (Bayer et al., 2007). These midbrain neurons project heavily to the striatum (Haber and Knutson, 2010), and in humans a striatal signal seen in functional magnetic resonance imaging (fMRI) data has been interpreted in terms of a prediction error signal as captured in computational models (O'Doherty et al., 2003; Seymour et al., 2004). Indeed, human positron emission tomography (PET) and pharmacological fMRI data support involvement of midbrain dopamine in prediction error signaling (Duzel et al., 2009; Pessiglione et al., 2006).

The temporal properties of the prediction error signal have sparked considerable controversy given their importance for understanding the genesis of this signal (Fiorillo et al., 2005; Redgrave et al., 1999; Redgrave and Gurney, 2006) but also in light of possible clinical implications. For example, in a previous study we found that prediction errors signals associated with reward anticipation are attenuated when participants expect the reward to be accompanied by an aversive consequence, a result that might account for altered decisions in depression (Talmi et al., 2009). An analysis of the associated time course could shed light on the degree of cognitive involvement in this effect. Unfortunately, the temporal properties cannot be studied using PET or fMRI due to their poor temporal resolution. Neuroimaging techniques that embody a better temporal resolution, such as electroencephalography and magnetoencephalography (M/EEG), are more appropriate for characterizing the temporal dynamics of prediction errors and can help integrate findings in humans with data from in vivo animal models. Although M/EEG are limited in their ability to detect signals from deep midline structures such as the striatum, they can readily detect local field potential fluctuations of cortical mass activity (with MEG being limited to tangentially oriented sources). Local field potentials are strongly modulated by afferent input and although there is no specific M/EEG signature of subcortical/cortical dopaminergic afferents, it is entirely possible that afferent prediction error signals arising from the midbrain could be detected with M/EEG.

The axiomatic model of reward prediction error (Caplin and Dean, 2008a,b) offers a formal test of whether a given neurobiological signal expresses a prediction error. Given that the MEG signal for prediction errors is not known, employing formal tests is crucial to avoid misinterpretation of outcome-locked responses that do not in fact code for prediction errors but might reflect other constructs such as valence, attention (salience) and outcome expectancy (Roesch et al., 2010; Rutledge et al., 2010). According to the axiomatic framework, any neurobiological signal which expresses prediction errors must comply with three axioms. First, signal magnitude should distinguish between better-than-expected events and worse-than-expected events, being either stronger or weaker for better-than-expected relative to worse-than-expected events. Second, signal magnitude should be proportional to the likelihood of events — the signal could be either weaker or stronger for likelier events relative to less likely events. Third, the magnitude of the signal should be equivalent for all fully-anticipated events.

Holroyd and Coles (Holroyd and Coles, 2002) have proposed that following a negative prediction error dopaminergic projections train the anterior cingulate cortex (ACC) to choose the best actions by disinhibiting the apical dendrites of motor-related neurons, and that the electrocortical difference between event-related potentials (ERPs) evoked in the ACC by positive and negative outcomes reflects prediction errors. Subsequent research extended this to positive prediction errors, suggesting that they inhibit the same dendrites (Nieuwenhuis et al., 2004). This electrocortical difference wave, termed Feedback-Related Negativity, FRN (Hajcak et al., 2006), typically peaks 230–270 ms after outcome onset, has a fronto-central maximum and its source has been localized to the medial prefrontal cortex (Gehring and Willoughby, 2002; Miltner et al., 1997). The N2 has also been found to express outcome valence and magnitude, and recent work suggests that the FRN may in fact be a modulation of the N2 (Holroyd et al., 2008; Kamarajan et al., 2009).

The FRN obeys the first requirement of the axiomatic model because its amplitude is determined from the difference between the ERPs elicited by unpredicted positive and negative feedback (Holroyd and Coles, 2002; Miltner et al., 1997). The FRN also expresses two other core features of a computational prediction error signal. First, a number of studies found that the FRN is sensitive to the likelihood of events (Holroyd et al., 2002, 2004, 2009; Holroyd and Krigolson, 2007). For instance, Holroyd and Krigolson (2007) showed that the FRN was larger when these outcomes were unexpected. Second, FRN has been shown to propagate back in time as a function of learning — initially time-locked to outcomes, but after learning solely expressed for predictive stimuli (Baker and Holroyd, 2009; Holroyd et al., 2002; Mars et al., 2005). For example, Baker and Holroyd (2009) showed that feedback indicating the absence versus presence of a reward in a maze-navigation task only elicited FRN when it was surprising. When the feedback was predicted by an earlier cue, however, it was the cue, rather than the feedback, which elicited FRN. The attenuation of the FRN when feedback was fully predictable is in line with the third axiom.

Notably, the sensitivity of FRN to outcome probability does not conclusively demonstrate that it complies fully with the second axiom. The second axiom requires that “better lotteries [with higher win probability] should always lead to lower dopamine release” (Caplin and Dean, 2008a, p. 197). Therefore, if the ERP signal associated with wins reflects dopamine release, it should be modulated by win probability, and the same should occur for losses (see Rutledge et al., 2010, Fig. 2B and discussion on p. 13,528). Demonstrating that the *difference* between positive and negative outcomes (the FRN) is sensitive to probability is therefore insufficient. One approach is to examine whether the average win and loss signals within the FRN time window are sensitive to probability. This approach was employed by Cohen et al. (2007), who found that outcome probability only modulated win outcomes, not loss outcomes. We employ the same approach here, to check whether the insensitivity of loss signal to probability in that study was driven by low power. However, we note that the separate win and loss signals are more susceptible to component overlap than the FRN difference wave (Luck, 2005), and thus should be interpreted with caution. In summary, research on the FRN provides strong evidence that this signal expresses prediction errors but fundamentally, the FRN cannot be tested for compliance with the second axiom. Furthermore, the axiomatic model makes the admittedly strict requirement that one study satisfies all three axioms, and despite an impressive volume of work no single dataset conforms to all of them. It is therefore possible that all existing datasets satisfy some of the axiomatic model's criteria, but violate others. Our secondary objective was to submit the EEG win and loss signals that form the FRN to the formal tests of the axiomatic model.

The magnetic equivalent of the FRN is not known, but there are indications that MEG is sensitive to errors and to feedback. An early study (Miltner et al., 2003) suggested that a magnetic equivalent of the ERN, a signal that follows the execution of errors and is thought to be closely related to FRN (Nieuwenhuis et al., 2004), can be detected and localized to the ACC, but the authors were not able to test their findings statistically. Three recent studies (Bayless et al., 2006; Mulas et al., 2006; Perianez et al., 2004) employed a set-shifting task akin to the Wisconsin Card Sorting Test, and compared ‘stay’ trials where the feedback indicated that responding on the basis of the set established in previous trials was correct to ‘switch’ trials where the feedback indicated that the set has shifted, so that participants' response was incorrect. These studies contrasted feedback-locked activation in the ‘stay’ and ‘switch’ conditions and found that the source of the difference between them localized to the ACC. Bayless et al. (2006) further established that the signal stemmed from the ‘switch’ trials and occurred 260 ms after feedback onset. It remains to be established whether this signal behaves like the FRN or obeys the framework of the axiomatic model.

To summarize, our primary objective here was to utilize MEG to identify a neurophysiological signal in humans that is consistent with all three axioms. We focused on MEG, because this neuroimaging modality may be less sensitive than EEG to component overlap, is more sensitive than EEG to detect neural activity arising from small foci (Oishi et al., 2002; Tao et al., 2005), and is less affected by the low pass properties of brain tissue and skull. Because the MEG signal for prediction error is not known, we employed an exploratory analysis approach: we filtered the data using a wide bandpass of 0.5–30 Hz and used SPM8 (Wellcome Trust Centre for Neuroimaging), which allowed us to extract group-level sensor-level signals consistent with the axiomatic model without prior assumptions about its spatial or temporal properties. EEG data was acquired simultaneously with MEG because if we obtain a classic FRN in the EEG modality we can be reassured that the processes our participants engaged with were similar to error processing in previous studies. Our secondary objective was to submit the EEG signal that comprises the FRN to the same analysis. In consideration of a vast prior literature and in contrast to our fully exploratory approach to MEG data, we employed a high-pass filter of 2 Hz to minimize the influence of the P300 and only examined the EEG signal that formed the FRN in frontal-central electrodes.

**Fig. 1:**
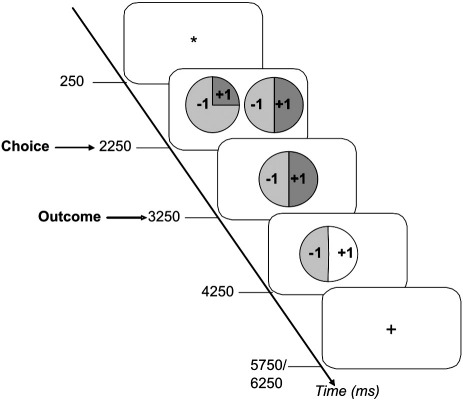
Timeline for a single trial in the gambling task. Participants viewed a fixation cross for 250 ms, and then were presented with a choice between two gambles. Gambles were presented in the form of pie charts with the probabilities of winning and losing indicated as the colored portion of the pie (here in shades of grey). The amounts to be won or lost were indicated on top of the relevant portion of the pie chart. Participants had up to 2250 ms to make their choice by pressing a key, and as soon as they had done so, the gamble they declined disappeared and the one they chose remained on the screen for 1000 ms. The outcome screen depicted an increase in luminance of the win or the loss portion of the pie (here in white), which indicated to participants whether they won or lost. All M/EEG data reported here are time-locked to the onset of the outcome screen, which remained visible for 1000 ms. The inter-trial interval then began, varying between 1500 and 2000 ms.

We scanned participants while they played a gambling game for real money where on each trial they were presented with a choice between two gambles. Target gambles were associated with a probability P (.25, .50, .75, 1) of winning £1, and a probability of 1-P of losing that amount. Note that this was not a learning task, as the probabilities of winning and the amounts that could be won were explicitly stated (Fig. 1). After participants selected the gamble they preferred, it was played according to a random process that conformed to the nominal probabilities. Gamble outcomes were revealed one second later and all analyses were time-locked to outcome onset.

**Fig. 2:**
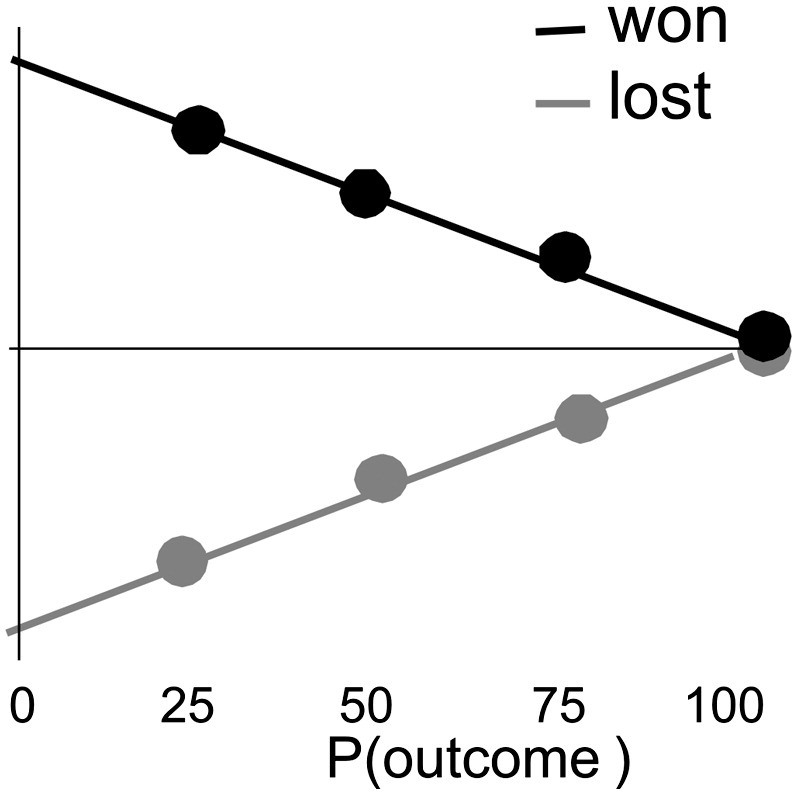
Theoretical predictions. The magnitude of the prediction error is depicted as a function of outcome probability, according to the predictions of the axiomatic model, with the additional assumption that participants prefer winning to losing. The largest prediction error occurs when outcomes are least likely (P = .25), with wins and losses generating prediction errors with opposite signs. When outcomes are known in advance (P = 1) the prediction error is smallest and win and loss signals are equivalent.

Fig. 2 depicts graphically the predictions of the axiomatic model for this experimental situation (Caplin and Dean, 2008a,b), with the additional assumptions that participants preferred winning to losing and that their baseline was keeping the initial endowment. The second axiom, that unexpected and expected events generate signals that differ in magnitude, requires that the absolute signal values of less likely events would be greater than those of more likely events, so that for example P = .25 events should have larger absolute signal than P = .50 events and those a large absolute signal than P = .75 events. This should be true both for wins and for losses. There are a number of ways to plot signal strength as a function of outcome probability according to this axiom. However, combining this axiom with the constraint that signals for wins and losses should differ for all uncertain (P < 1) outcomes (first axiom), and that the signals for fully-anticipated (P = 1) wins and losses should be equivalent (third axiom), implies that the slopes of these probability functions for win and loss outcomes should be significantly different from zero, different from each other, and have opposite signs. These are minimal requirements for any prediction error signal. We therefore tested for electromagnetic signals which (a) exhibited a significant interaction between outcome valence and outcome probability (b) exhibited significant simple effects of probability within both win and loss conditions, and (c) which remained significant after masking out any activation that differed between the fully-anticipated win and lose conditions.

## Materials and methods

### Participants

17 right-handed, healthy adults (mean age 22.29 years, SD = 3.25, 10 females), participated in the study and were compensated for their time according to their actual winning in the gamble task (see procedure). Participants were screened for psychiatric and neurological history. The study was approved by the UCL ethics committee.

### Materials

In each trial, participants chose between a target gamble and a lure gamble. Each gamble was presented in the form of a pie chart with the probability of winning being equivalent to the portion of the circle filled with the win color and the probability of losing equivalent to the portion of the chart filled with the lose color (De Martino et al., 2006). The win and lose colors were blue or yellow, and color assignment to condition was randomized across participants. The amounts participants could win or lose were displayed as a number on top of the relevant portion of the chart (+£1, −£1, +£2, −£2). The lure gamble was drawn randomly in each trial with the constraint that it would have a lower expected value than the target gamble. Target gambles were always associated with a £1 amount, and belonged to one of eight cells according to an outcome valence (win, loss) by outcome probability (.25, .50, .75, 1) design. To ensure that participants experienced approximately 45 target gambles in each of the 8 design cells, we presented participants with 45 gambles with 0 or 1 win probabilities, 90 gambles with a 50% win probability, and 180 gambles with 25% or 75% win probabilities, a total in all of 540 experimental trials. A weighted random sampling process ensured that actual outcome probabilities matched the nominal win probabilities.

### Procedure

#### Behavioral procedure

Participants were read detailed instructions of the experiment and subsequently gave written consent to participate in the study. Participants received 40 pounds before they began the experiment. They were instructed to conceal this amount in their bag or jacket pocket (which was stored securely outside the scanner room) and told that this amount was theirs to keep and would be topped up by any amount they win, but equally they would have to pay the experimenter back any amount they lose. Wins and losses were capped to £24, so final compensation ranged between £16 and £64, a range which represented a 70% confidence interval around the average compensation of £40. The cumulative winnings or losses were displayed at the end of each block. There were 9 experimental blocks, with 60 trials in each. Each block included the same number of target gambles of each win/lose probability, and trial order was random.

Participants also took part in a long practice trial to give them an experiential ‘feel’ for these probabilities (Hertwig and Erev, 2009). A practice block, identical to the experimental block was given first. Here the win color was always green, the loss color always red, and participants were told that the amounts represented pennies instead of pounds, and that their initial endowment was 20 pennies instead of £40. To emphasize to the participants that they were playing with real money they were given an envelope with twenty 1-penny coins before the practice block. Following practice the experimenter visibly added coins to the envelope, or removed them, according to the amount participants won or lost in the practice block.

Each trial began with a fixation cross that was displayed for 250 ms. The choice screen followed and displayed target and lure gambles. A single gamble was presented on the left and right side of the screen. The, with positions of target and lure gambles were assigned randomly in every trial. Participants indicated which gamble they preferred by pressing one of two keys with their left or right thumbs. They were further instructed that if they failed to respond the computer would make a choice for them, and further, that the computer would always pick the worst gamble. The choice screen remained until participants made a choice, or until 2 s elapsed. The non-preferred gamble then disappeared from the screen and the preferred gamble was displayed for 1000 ms. The outcome screen then announced the result of the gamble as a change in the luminance of the relevant portion of the circle. For example, when the ‘win color’ was blue, the blue portion brightened to signify a win or the yellow portion brightened to signify a loss. The outcome screen was displayed for 1000 ms and replaced by a crosshair signaling the inter-trial interval, which varied randomly in duration between 1500 and 2000 ms.

#### MEG procedure

##### MEG recordings

MEG data was recorded using a 275-channel CTF Omega system whole-head gradiometer (VSM MedTech, Coquitlam, BC, Canada). Neuromagnetic signal was continuously recorded at 600 Hz sampling rate and low-pass filtered online at 120 Hz. After participants were comfortably seated in the MEG, head localizer coils were attached to the nasion and 1 cm anterior of the left and right tragus to monitor head movement during recording.

##### MEG data analysis

Event-Related Fields (ERFs) were time-locked to the feedback stimuli with analyses implemented within SPM8 (Kiebel and Friston, 2004a,b) and MATLAB 7 (The MathWorks, Inc., Natick, MA, USA). Data were down-sampled to 200 Hz offline, and filtered with a Butterworth filter between .5 and 30 Hz. This wide bandpass filter was used to maintain an exploratory approach to the data while avoiding drift across trials. Epoched MEG data was generated for a period 160 ms prior to outcome onset until 700 ms after outcome onset. The negative part of the time axis was used as baseline. Trials with artifacts exceeding 1.5 ∗ 10^−10^T were removed automatically and additional artifactual trials were removed following manual inspection (0.4% of all trials, fewer than 4% of trials for any individual participant). Eye blink confounds were corrected by a signal space projection (SSP) method (Nolte and Hamalainen, 2001) implemented in MEEGTools toolbox distributed with SPM8. Artifact subspace was defined per session by principal component analysis of session-averaged data epoched around eye blinks, using subject-specific thresholds for blink detection and 4 principal components (numbers which were established empirically to optimize confound removal) and 4 principal components.

**Fig. 3:**
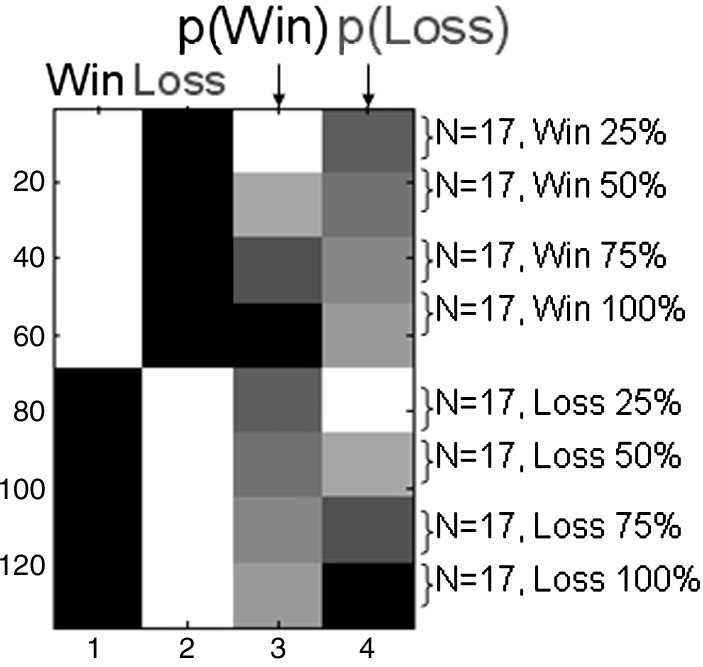
MEG design matrix. The design matrix shows the 2nd level analysis of covariance. Images corresponding to each cell of the 2 (outcome valence: win, loss) by 4 (outcome probability: .25, .50, .75, 1) design were entered into the model for each subject. The two win and loss regressors were modulated linearly by outcome probability using the values [4, 3, 2, 1, 0 0 0 0] and [0 0 0 0 4 3 2 1]. SPM automatically subtracts the mean from each parametric modulator to center it around zero.

The SPM analysis proceeded in two steps. First, for each subject and condition, a 3D channel space by time was created by projecting, for each sample point, the sensor locations onto a plane following by a linear interpolation to a 64 × 64 pixel grid (pixel size = 3 × 3 mm). These images were smoothed using a Gaussian kernel Full Width Half Maximum (FWHM) of 8 mm/ms. Second, these images were masked temporally between 100 ms and 600 ms and entered into an ANCOVA design. Each of the ‘Win’ and ‘Loss’ regressors were modulated parametrically by a linear covariate corresponding to outcome probability (.25, .50, .75, 1; see Fig. 3).

We explored the main effect of *outcome valence* with an F test of the contrast [1–1 0 0] between the Win and Loss regressors. We explored the main effect of *outcome probability* with an F test [0 0 1 1] over both win and loss probability covariates. This test exposes a signal that covaries with outcome probability in the *same* direction in *both* win and loss conditions by extracting regions where the sum of the slopes of the functions relating win and loss ERFs to their outcome probabilities is significantly different from zero. Finally, we examined the interaction between outcome valence and outcome probability with an F test of the contrast [0 0 1–1] between the Win and the Loss probability covariates. This test reveals regions in which the slope of the MEG signal as a function of win probability is different from the slope of the MEG signal as a function of Loss probability, as required from a prediction error signal according to the axiomatic model. All F-tests were statistically thresholded with peak-level threshold of P = 0.005 (F = 11.37), uncorrected, following Bunzeck et al. (2009). Only clusters exceeding an extent of 100 mm/ms are reported to minimize false positives. Finally, we entered images corresponding to the fully predictable outcomes in the win and the loss conditions (P = 1) as two regressors in a separate 2nd-level design matrix. We used an F contrast to reveal ERFs that differed significantly between fully predictable wins and losses with a lenient statistical threshold (P < .05), and created a corresponding map of sensitivity. We then inverted the sensitivity map with the Imcalc tool in SPM8, so that it only included regions where amplitude changes did not differ significantly between fully predictable outcomes. We used this map as an inclusive mask for the analysis of the critical interaction effect.

#### EEG procedure

##### EEG recordings

EEG data were concurrently acquired with MEG. EEG data were recorded from the scalp using Ag/AgCl electrodes located at 11 standard positions (AF7/8, AFz, Fz, Cz,Pz, P3/4, M1/2) mounted in an electrocap (Electro-Cap, International 10–20 system locations). Vertical eye movements were monitored with electro-oculogram (EOG) sensors placed 2 cm above and below the outer canthus of the left eye. All EEG sensor activity (11 electrodes) was referenced online to Cz, digitized at a rate of 480 Hz and filtered online with a bandpass of 0.01–120 Hz. Electrode impedances were kept below 10 kOhm. Offline, data were analyzed using SPM8. The electrophysiological signals were re-referenced to the mean of the activity at the two mastoids processes (M1/2), down-sampled to 200 Hz and filtered with a Butterworth filter between 2 and 30 Hz; a high-pass filter of 2 Hz was chosen to minimize the influence of P300 on detecting the FRN. Outcome-locked ERPs were computed for a period 160 ms prior to outcome onset until 700 ms after outcome onset. Single-trial ERPs were averaged separately for each of the 8 probability and valence conditions using the ‘robust averaging’ method in SPM8. This is a simple special case of the robust general linear model (Litvak et al., 2010; Wager et al., 2005). For each channel and time point the distribution of values over trials is considered and the outliers are down-weighted when computing the average. This makes it possible to neutralize artifacts restricted to narrow time ranges without rejecting whole trials. Moreover, a clean average can be computed with no clean trials, given that the artifacts do not consistently overlap and only corrupt (different) parts of trials. Averages were then low-pass filtered at 30 Hz to remove high frequencies introduced by the robust averaging method.

##### EEG data analyses

We analyzed the EEG data in two ways. First, following an established methodology in FRN studies (Holroyd and Krigolson, 2007), we analyzed the difference waveform by subtracting win from loss trials at Cz electrode, and identified the FRN as the maximum negativity within the window of 0–600 ms after feedback onset. Second, in keeping with other FRN studies and with the rationale of the axiomatic model, ERPs for all 8 conditions were averaged from Cz across the time window of 200–300 ms, selected on the basis of previous work (Gehring and Willoughby, 2002; Marco-Pallares et al., 2008) to minimize potential noise fluctuations in these waveforms.

## Results

### Behavioral results

Participants chose the target gamble 99% of the time. The frequency of choosing the target gamble [F(4,64) = 3.27, P < .05] and choice latency [F(4,64) = 30.03, P < .001] varied across the five target gamble win probabilities (0, .25, .50, 75, 1), with participants slowest when forced to choose a target gamble that led to certain loss (see Table 1). These effects should not influence the results because ERFs and ERPs were studied time-locked to the onset of the outcome screen, a full second after a key-press indicating actual choice.

### MEG results

#### Outcome valence

**Fig. 4:**
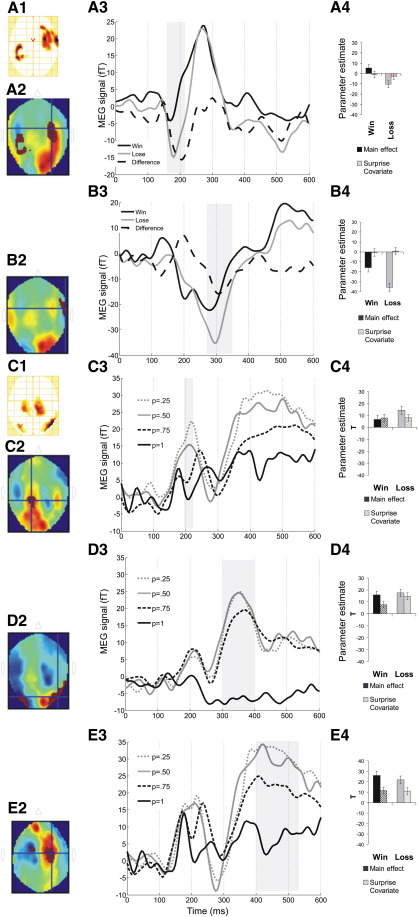
MEG signals expressing the main effects of outcome valence (A, B) and outcome probability (C, D, E). (A1, C1). SPMs depicting the main effects of valence (A1) and probability (C1), overlaid on the glass brain. (A2–E2). SPMs depicting the main effects are depicted in dark red, with the crosshair placed on the significant peak within a cluster. The main effect of valence (wins > losses) is overlaid on the mean-corrected average signal for wins, with warm colors corresponding to positive and cold colors to negative signal amplitude relative to baseline. Valence was associated with significant amplitude change 190 ms (A2) and 310 ms after outcome (B2). The main effect of probability (win probability-loss probability) is overlaid on the mean-corrected average signal for win probability. Again, warm colors correspond to positive and cold colors to negative signal amplitude relative to baseline. Probability was associated with significant amplitude change 205 ms (C2), 340 ms (D2) and 425 ms (E2) after outcome. A3–E3. Average ERFs (in fento-Tesla units) extracted from the crosshair locations in panels A2–E2, respectively. A4–E4. Parameter estimates for wins (blue), losses (red), and the covariates representing their modulation by probability (hashed) for the peaks in panels A2–E2.

This analysis revealed two clusters, right and left lateralized, which expressed the difference between wins and losses with maxima 190–220 ms following outcome onset. The signal amplitude for losses was higher than that for wins in the left-lateralized cluster (earliest maximum 200 ms after outcome, Z = 3.39, at x = − 30, y = − 44, k = 805^1^), and the signal amplitude for wins was higher than that for losses in the right-lateralized cluster, depicted in Fig. 4A (earliest maximum 190 after outcome, x = 32, y = 8, k = 1236). For axial gradiometers as used in our MEG system, bilateral dipolar scalp topography suggests a source in the middle of the positive and negative peaks. Fig. 4A3 shows that the time course extracted from this peak resembled the FRN, but the parameter estimate plot reveals that the signal was not sensitive to outcome probability. Fig. 4B depicts two other right-lateralized clusters, which expressed the difference between wins and losses at a single location but at two different time points. The earliest maximum was 115 ms after outcome onset (x = 66, y = 2, k = 279) and the later maximum was 310 ms after outcome onset (x = 64, y = 5, k = 814). Finally a fifth cluster expressed outcome valence 410–500 ms after outcome onset (x = 26, y = 40, k = 1300, data not shown).

#### Outcome probability

This contrast revealed five clusters of activation, two of which are depicted in Fig. 4C and D. The earliest expression of probability, depicted in Fig. 4C, was 205 ms after outcome onset (x = − 13, y = − 27, k = 166). Although the parameter estimate plot shows that this cluster was also sensitive to outcome valence, this effect was not statistically significant. Two posterior clusters, left- and right-lateralized, expressed outcome probability 340 ms following outcome onset with a negative and a positive sign, respectively (left-lateralized: x = − 34, y = − 76, k = 1074 right-lateralized: x = 47, y = − 70, k = 1990), suggesting a posterior midline source. The right-lateralized cluster is depicted in Fig. 4D. Two more anterior clusters, left- and right-lateralized (Fig. 4E), expressed outcome probability 395–520 ms following outcome onset with a positive and a negative sign (left-lateralized: maximum 425 ms after outcome, x = − 23, y = − 22, k = 1545; right-lateralized: maxima 395 ms after outcome, x = 13, y = − 22, k = 1344 and 520 ms after outcome, x = 15, y = − 6, k = 1074), suggesting a more anterior midline source.

#### Interaction

**Fig. 5:**
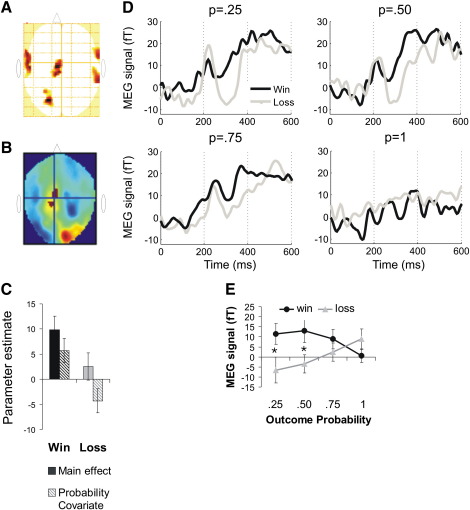
The MEG signal expressing the interaction between outcome valence and probability. (A) SPM for the interaction effect, overlaid on the glass brain. (B) An SPM for the interaction effect 320 ms after outcome, overlaid on the average signal for win probability. Warm colors correspond to positive and cold colors to negative signal amplitude relative to baseline. The crosshair is placed on the location where win and loss probability slopes were significantly different from each other and from zero, but the 100% certain wins and losses did not differ from each other significantly. (C) Parameter estimates for wins (blue), losses (red), and the covariates representing their modulation by probability (hashed) for the peak in panels B. (D) Average ERFs (in fento-Tesla units) for wins and losses, plotted separately for each outcome probability, extracted from the peak in panel B. (E) Average ERFs across a 50 ms time window around the peak activation in panel B, plotted separately for wins and losses as a function of outcome probability. Paired-sample t-tests demonstrated that the difference between wins and losses was significant when outcome probability was 25% or 50% but not when it was higher.

This key analysis was taken to identify a MEG prediction-error signal and was limited to maxima where amplitude changes did not distinguish between fully-predictable wins and losses. The interaction contrast revealed a number of clusters (Fig. 5A) but only in two maxima was evoked activity sensitive to *both* win and loss probabilities. Fig. 5 shows a midline cluster (x = − 9, y = − 9, k = 362) where the slopes of evoked activity for wins and losses as a function of probability 320 ms after outcome had opposite signs, in line with the requirements of the axiomatic model. Strikingly, the time course extracted from the peak of this cluster resembled the FRN (Fig. 5D).

To enable a direct comparison with the EEG data, we averaged the MEG data over the period of 295–345 ms following outcome onset and entered these averages into a repeated-measures ANOVA with the factors outcome probability and outcome valence. The main effect of valence was significant, F(1,16) = 5.85, P < .05, but was qualified by a significant interaction with probability, F(3,48) = 6.74, P = .001. The main effect of probability was not significant, F < 1. Post-hoc polynomial tests confirmed that probability linearly modulated the difference between the win and loss signals, F(1,16) = 15.06, P = .001. However, a series of paired t-tests comparing P = .25 to P=. 50, P = .50 to P = .75, and P = .75 to P = 1 conditions did not yield any significant results, P > .05. A series of paired t-tests comparing wins and losses demonstrated that wins and losses differed in the P = .25 [t(16) = 3, P < .01] and P = .50 [t(16) = 3.39, P < .01] condition but not in the P = .75 and the P = 1 conditions, P > .05. Thus, this magnetic scalp signal obtained here can be thought of as weakly satisfying the axiomatic criteria (Rutledge et al., 2010). For the second cluster identified in this analysis (x = − 57, y = − 9, k = 375), 100 ms after outcome onset, no post-hoc t-tests were significant and it was not analyzed further.

### EEG results

#### Difference wave analysis

Here FRN magnitudes were computed per subject by subtracting wins from losses. The minimum of the difference waveform within the interval of 600 ms following feedback onset defines the FRN. These minima were entered into a one-way repeated-measures ANOVA of outcome probability (P = 0.25, 0.5, 0.75, 1). The main effect of probability was significant [F(3,48) = 10.17, P < .001], and varied linearly and as a function of probability [F(1,16) = 17.40, P = 0.0.1], replicating previous results.

## Average time window analysis

**Fig. 6:**
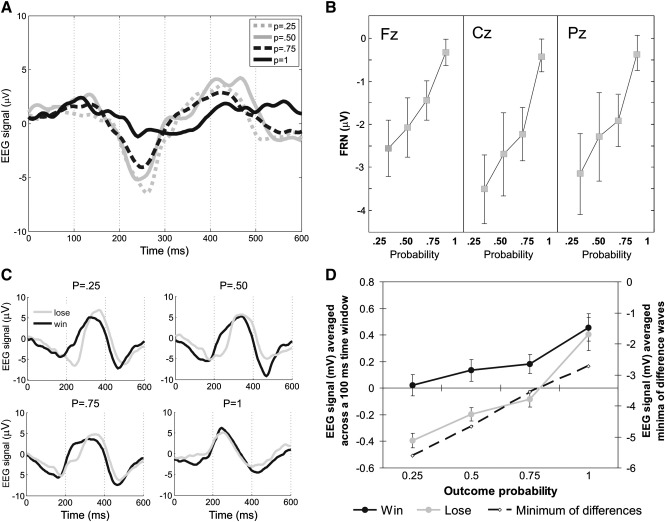
Feedback-locked EEG signal. (A) Grand average ERPs obtained by subtracting win from loss trials for each probability condition at Cz EEG electrode. (B) Grand average FRNs calculated using average time window analysis for each probability condition at Fz, Cz, and Pz electrodes. (C) Grand average ERPs for win (black) and loss (grey) trials for each probability condition. (D) Grand averages for the EEG signal computed in two different ways. Solid lines: results from the average time window analysis showing the average signal for wins (black) and losses (grey), for each one of the probability conditions. Dashed line: results from the difference-wave analysis. Error bars represents standard error of the mean.

Averaged ERPs across the time window 200–300 ms were entered into repeated-measures ANOVA with the factors outcome probability and outcome valence. Both main effects were significant [valence: F(1,16) = 30.05, P < 0.001; probability: F(3,48) = 20.67, P < 0.001], as was their interaction [F(3,48) = 5.32, P < .05]. The difference between the signal magnitudes for wins and losses was smaller for more likely outcomes. These results indicate that we obtained a significant FRN, which was modulated by probability, replicating previous findings. However, examination of Fig. 6 shows that both win and loss signals varied *positively* with outcome probability, in contradiction with the requirements of the axiomatic model that the sign of the slope for wins would be opposite to the sign of the slope for losses.

## Discussion

The axiomatic model of reward prediction errors prescribes that any neural signal expressing a prediction error must comply with a set of three formal requirements. We explored the magnetic sensor-space for a brain signal that complied with those, and specifically searched for a signal that expressed the interaction between win and loss probabilities. The interaction contrast isolated a fronto-central MEG signal that peaked 320 ms after outcome onset and complied reasonably well with the axiomatic model. This signal varied significantly as a function of outcome probability both when the outcome was a win and when it was a loss, indicating that it differentiated more and less likely events. Wins correlated negatively with outcome probability and losses correlated positively with outcome probability, and these correlations were significantly different from each other. This indicates that highly unlikely wins and losses generated the strongest absolute signal values, and that highly likely wins and losses generated the smallest absolute signal values. This signal also complied with the final axiom that the P = 1 wins and losses would not differ. We conclude that we have successfully indentified, for the first time, a MEG signature of reward prediction error expressed by the interaction MEG signal. Its peak time resembled that of an EEG prediction error signal obtained using an innovative single-trial modeling approach (Philiastides et al., 2010).

The final axiom corresponds to a null effect, which means it is never possible to test it empirically. Any study can only attempt to give assurances that power was sufficient to detect real differences. Our data provides two such assurances. Firstly, our analysis of the interaction effect employed a conservative statistical threshold of P < .005, but we employed a more lenient statistical threshold of P < .05 to detect differences between fully-anticipated (P = 1) wins and losses. The regions identified in that contrast were subsequently excluded from the interaction analysis. Second, the waveform for uncertain (P < 1) outcomes differed from the waveform for certain (P = 1) outcomes in two ways, suggesting different neural computations. First, in P < 1 conditions the win signal was more positive than the loss signal, but this difference flipped for the P = 1 outcomes. Second, while the time course from the uncertain conditions bore a striking resemblance to the classic fronto-centrally distributed FRN, this was no longer the case for the certain, P = 1 outcomes, which appeared to only reflect noise. If that was true, it would fully conform to a major tenet of reinforcement learning theory, which states that the prediction error associated with P = 1 outcomes would be expressed when participants realize what these outcomes are going to be, rather than at the time of outcome revelation. In our paradigm the prediction error signal associated with participants' realization that they were about to win or lose a certain amount for sure must have been expressed earlier in the trial, after the presentation of the two gambles but prior to the key-press response that indicated the end of the comparison process. Although the complex choice and comparison process which took place prior to outcome revelation prevented us from analyzing the signal associated with the predictive gamble stimuli, single-cell recordings demonstrate cue-locked but not outcome-locked dopamine firing when the cue fully predicted the outcome (Schultz et al., 1997; Schultz and Dickinson, 2000).

The claim that the interaction signal we obtained expresses a prediction error should be treated with caution because we did not have sufficient power to test all the detailed predictions of the axiomatic model (Caplin and Dean, 2008b). Future research should take steps to ensure sufficient power to test these, possibly by using multivariate integration across sensors (Friston et al., 1996), an approach we are currently exploring. Finally, because we used a statistical threshold which was not corrected for multiple comparisons, future research will be necessary to corroborate our result.

Magnetic effects of probability and valence were observed before the emergence of the prediction error signals, 200 ms after outcome. The early valence effect is also in marked agreement with the EEG data of Philiastides et al. (2010) who also found that this signal preceded the prediction error signal. These authors suggested that prediction error processing proceeds in multiple stages, with scalp measurements expressing the components of the prediction error signal before the fully integrated signal can be detected. Additionally, we observed a later probability signal, 340 ms after outcome onset, which resembled the P300, the electrophysiological signal most strongly associated with coding uncertainty in many settings (Donchin, 1981; Sutton et al., 1965), including surprise in gambling tasks (Hajcak et al., 2005, 2007).

The axiomatic model provided a solid framework with which to scrutinize the MEG signal, but also offered a novel approach to the analysis of EEG signal most closely associated with prediction errors, the FRN. Although many datasets show that the FRN complies with the requirements of the axiomatic model, none show that it complies with all of them. This admittedly strict requirement merited an analysis of the EEG dataset in light of the axiomatic model.

We obtained a robust FRN using simultaneously acquired EEG, with a peak time of 270 ms post-outcome, which is compatible with previous literature (Holroyd and Coles, 2002; Miltner et al., 1997). This ERP was sensitive to outcome probability and reduced almost to zero when gamble outcomes were fully known in advance (P = 1), replicating previous studies (Baker and Holroyd, 2009; Holroyd et al., 2002, 2004, 2009; Holroyd and Krigolson, 2007; Mars et al., 2005). Examining wins and losses separately, however, revealed that although both were sensitive to outcome probability, in line with the axiomatic model and extending the results of Cohen et al. (2007), they varied with probability in the same direction, at odds with the requirement for bidirectional signaling embodied in the axiomatic model (Caplin and Dean, 2008a,b). This requirement stems from the known characteristics of the computational prediction error signal (Sutton and Barto, 1998), which is symmetrical for positive and negative prediction errors. The computational signal is compatible with evidence that phasic activations of dopaminergic neurons reflect positive prediction errors while a pause in firing is thought to reflect negative prediction errors equally strongly (Bayer et al., 2007). When we analyzed the average EEG signal for wins and losses within our specified time window our data revealed that the ERPs that formed the FRN were more negative for unlikely wins – where *most* dopamine is released – than likely wins, suggesting that these ERPs correlate inversely with dopamine release. Yet at the same time, the ERPs were also more negative for unlikely losses – where *least* dopamine is released – than likely losses, suggesting the very opposite.

A vast literature supports the suggestion that the difference waveform FRN expresses prediction errors, but the axiomatic model requires separate analysis of the win and loss signals, and does not lend itself directly to an analysis of the difference waveform. Taken at face value, our data shows that the EEG signal at the time that the FRN is detected does not comply with the interpretation of the FRN as a direct marker of dopaminergic prediction error. One interpretation is that the striatal training projections to the anterior cingulate cortex are not a one-to-one reflection of the prediction error but instead reflect an additional computational step in the service of future behavior optimization (Holroyd and Coles, 2002). Another interpretation is that the FRN correlates with another component of error processing (Roesch et al., 2010). Finally, it is also possible that ERP components which co-occurred at the same time as the FRN masked the true nature of the separate win and loss signals. The fact that the MEG signal satisfied the axiomatic test and the EEG signal did not could be due to differential degrees of overlap with other components in the two brain signal recording modalities. Future model-based analyses of M/EEG data should explicitly model brain responses that overlap with the signal of interest.

The current study obtained two MEG signals which resembled the FRN. The MEG interaction signal resembled the FRN in its waveform, and expressed probability in a way that conformed to the axiomatic model's criteria for a prediction error signal. Additionally, an early valence effect also resembled the FRN in its waveform, although it was not sensitive to outcome probability. Interestingly, the same early valence signal was also found in EEG by Philiastides et al. (2010), who employed an EEG trial-by-trial modeling approach.

The variability in the sensitivity of FRN-like signals to probability mimics the conflicting evidence for the effect of probability on FRN in previous studies, where it is often (Holroyd et al., 2003; Yasuda et al., 2004) but not always (Hajcak et al., 2005) obtained, and where probability may modulate positive outcomes, negative outcomes, or the difference between them. It has been suggested that this effect depends on the degree to which the actions of participants actually cause the outcome (Holroyd et al., 2009), and on the way participants perceive the action-outcome contingencies (Hajcak et al., 2007). A sense of involvement in the task has been shown to correlate positively with FRN amplitude (Yeung et al., 2005). Here participants selected the gamble they played and had complete information about the contingencies, but their action did not meaningfully influence whether they would win or lose. The influence of probability on FRN-like signals in the current study suggests that participants felt sufficiently involved in the task, perhaps because they were endowed with a large sum of money (£40) which they knew they'd have to pay back if they lost. The endowment effect (De Martino et al., 2009; Knutson et al., 2008) may have led our participants to care more about gamble outcomes than in other studies. Yet even though the MEG interaction signal and the FRN in the EEG data were sensitive to probability, the early FRN-like signal was not.

M/EEG studies typically focus on how known components are modulated by experimental manipulations. This approach is not optimal when there are no established correlates of the phenomenon of interest, such as the MEG correlates of prediction errors. This approach is also less informative when multiple electrophysiological markers may be involved. Indeed, previous work implicates a number of different components in the representation of outcome valence, surprise, and magnitude, such as the FRN, N2, and P300 (Hajcak et al., 2005, 2007; Holroyd et al., 2006, 2008; Kamarajan et al., 2009; Sato et al., 2005; Yeung et al., 2005; Yeung and Sanfey, 2004). When research questions warrant an exploratory analysis, as is the case here, the SPM approach provides an elegant solution.

An exploratory analysis of complex datasets, such as those obtained by neuroimaging, is more likely to succeed when a research question is tightly operationalized. We were able to do so here by invoking the framework of an axiomatic model, which provides a well specified series of tests that any neurobiological signal expressing prediction errors must comply with. By contrast, fitting the predictions of temporal difference error models to imaging data is fraught with issues stemming from the use of highly parameterized assumptions in combination with a regression approach (Caplin and Dean, 2008a,b; Roesch et al., 2010; Rutledge et al., 2010). This is even more challenging in the case of M/EEG data, where model-based approaches require a trial-by-trial analysis technique (Mars et al., 2008), a challenge at the forefront of M/EEG research (Philiastides et al., 2010). By using SPM to test the axiomatic model's predictions we benefited from its formality but suffered none of the usual disadvantages of a model-based approach.

## Conclusions

Many ERP studies have set out to explore a prediction error signal, but its exact nature is still controversial. By using the stringent criteria of an axiomatic model we have identified, for the first time, a corresponding MEG signature of a human prediction error signal emerging approximately 320 ms after an outcome. The ERFs associated with wins and losses varied with outcome probability in a bidirectional manner, in line with the axiomatic model. The striking resemblance between this signal and the FRN provides converging evidence to claims that FRN expresses a prediction error (Holroyd and Coles, 2002). Although we queried interpretations of the FRN in our EEG data in light of the axiomatic model, the fact that the same waveform expressed prediction errors in our MEG dataset, suggests that further research is needed to understand fully the exact nature of this signal. Our approach opens the possibility to further characterize computational models of prediction errors in humans non-invasively and to examine explicitly the critical issue of timing embodied in such signals.

## Figures and Tables

**Table 1:** 

Win probability		0.00	0.25	0.50	0.75	1.00
Choice of target gamble (%)	mean	98.95	99.61	99.41	97.91	99.35
SD	1.59	0.58	0.97	2.59	1.31
Latency (ms)	mean	662.53	522.66	538.29	563.85	540.03
SD	112.68	90.91	113.26	117.21	103.41

## Footnotes

1 According to convention, the origin of the X and Y axes is at the center of the brain, and k represents spatial extent in mm.
